# Outcomes prediction in pre-operative radiotherapy locally advanced rectal cancer: leucocyte assessment as immune biomarker

**DOI:** 10.18632/oncotarget.25023

**Published:** 2018-04-27

**Authors:** Alexis Vallard, Max-Adrien Garcia, Peng Diao, Sophie Espenel, Guy de Laroche, Jean-Baptiste Guy, Majed Ben Mrad, Chloé Rancoule, David Kaczmarek, Thierry Muron, Gregoire Pigné, Jack Porcheron, Michel Peoc'h, Jean-Marc Phelip, Julien Langrand -Escure, Nicolas Magné

**Affiliations:** ^1^ Department of Radiation Oncology, Lucien Neuwirth Cancer Institute, Saint Priest en Jarez, France; ^2^ Public Health Department, Hygée Institute, Saint Priest en Jarez, France; ^3^ Department of Radiation Oncology, Sichuan Cancer Hospital, Chengdu, Sichuan Province, China; ^4^ Department of Thoracic and Digestive Surgery, Private Loire Hospital (HPL), Saint Etienne, France; ^5^ Department of Medical Oncology, Lucien Neuwirth Cancer Institute, Saint Priest en Jarez, France; ^6^ Department of Digestive and Oncologic Surgery, North University Hospital, Saint Priest en Jarez, France; ^7^ Department of Pathology, North University Hospital, Saint Priest en Jarez, France; ^8^ Department of Hepatology and Gastroenterology, North University Hospital, Saint Priest en Jarez, France

**Keywords:** rectal cancer, chemoradiation, neutrophil, lymphocyte, ratio

## Abstract

**Objective:**

Leukocytes are hypothesized to reflect the inflammatory tumor microenvironment. We aimed to validate their prognostic significance in a large cohort of patients treated with pre-operative radiation for locally advanced rectal cancer (RC).

**Results:**

From 2004 to 2015, 257 RC patients with available biological data underwent a pre-operative radiotherapy, with a median age of 66 years. The median rectal EQD2 was 49.2Gy. Most of patients experienced concurrent chemotherapy (*n* = 245, 95.4%), mainly with 5-FU (83.3%). Clear surgical margins (i.e. complete resection) were achieved in 234 patients (91.1%). A complete (Mandard TRG1: *n* = 35, 13.6%) or almost complete pathological response (Mandard TRG2: *n* = 56, 21.8%) were achieved in 91 patients (35.4%). With a median follow-up of 46.1 months, 8 patients (3.1%) experienced local relapse, 38 (14.8%) experienced metastases and 45 (17.5%) died. Elevated pre-radiation neutrophil to lymphocyte ratio (NLR > 2.8) was identified as an independent predictive factor of increased local relapse, of decreased progression-free survival and overall survival in multivariate analysis. Elevated NLR was marginally associated with incomplete pathological response in multivariate analysis, suggesting a possible value as a biomarker of radio-sensitivity.

**Conclusions:**

Pre-radiation NLR is a simple and robust biomarker for risk stratification in locally advanced RC patients undergoing pre-operative radiotherapy, and might select the subpopulation eligible to treatment intensification or to neoadjuvant chemotherapy.

**Material and Methods:**

Clinical records from consecutive patients treated in a single institution between 2004 and 2015 with curative-intent radiotherapy were retrospectively analyzed. Classical prognosis factors of RC and peripheral immune markers based on lymphocytes and neutrophil counts were studied.

## INTRODUCTION

In a recent meta-analysis, the 7-year overall survival (OS) rate of stage II/III locally advanced rectal cancer (RC) patients was 62% despite optimization of the treatment planning process and integration of systemic agents to improve treatment efficacy [1]. Currently identified prognosis factors for survival (T stage, age, grade, number of examined lymph nodes and number of positive nodes [2]) are therefore not discriminant enough to detect patients with the highest risks of tumor relapse. Identification of new biomarkers is crucial in order to isolate the subpopulation that might require treatment intensification. Unlike promising but expensive biomarkers based on genetic profiling [3, 4], leukocyte and neutrophil counts are already performed in daily routine, making them accessible regardless of the local technological development. Interestingly, there is a strong rationale suggesting that systemic inflammatory response to tumors is manifested in the peripheral white blood cells [5–7]. In various primary tumor locations, peripheral immune markers (leukocytosis, neutrophilia, neutrophil to lymphocyte ratio (NLR)) were recently suggested to be possible biomarkers of radiosensitivity, predicting local relapse and survival in patients treated with definitive chemoradiation [5, 7, 8]. The NLR was suggested to be an independent prognostic factor for recurrence and mortality in large cohorts of colorectal cancer patients [9, 10] and in small cohorts of RC patients [11–15]. However, to our best knowledge, the impact of peripheral immune markers on prognostication has never been assessed specifically in a large cohort of locally advanced RC patients.

The aim of the present study was to analyze whether peripheral immune markers of locally advanced RC patients was associated with local tumor control and global outcome.

## RESULTS

### Patient, tumor and treatment characteristics

A total of 477 consecutive metastasis-free RC patients underwent a pre-operative radiotherapy between 2004 and 2015 at the institute, with 220 exclusions because of missing biological data. Thus, data of 257 patients were analyzed in the present study. At time of radiotherapy, median age was 66 years (IQR: 56.4–73), with 170 males and 87 females. Patients were in good condition, with 251 patients (97.7%) with an WHO Performance Status 0–1. Most of RCs (*n* = 239, 93%) were diagnosed at a locally advanced setting (stage II–III). Tumors were located in the lower (*n* = 114, 44.4%), middle (*n* = 117, 45.5%) or upper (*n* = 26, 10.1%) rectum. Pathological analyses identified adenocarcinoma (*n* = 257, 100%), mainly with moderate differentiation (*n* = 126, 49%). Vascular invasion was identified in 56 (20.2%) tumors. The baseline median lymphocyte count was 1.81 × 10^9^/L (IQR: 1.45–2.21). The baseline median neutrophil count was 4.05 × 10^9^/L (IQR: 3.17–5.35). There was no statistical correlation between the baseline median neutrophil count and the tumor stage (*p* = 0.06). All patients underwent a preoperative radiotherapy, mainly based on 3D-conformational radiotherapy (*n* = 243, 94.5%), without rectal dose reduction (*n* = 130, 50.6%). Median rectal EQD2 was 49.2 Gy (IQR = 43.9–50), with a median radiation duration of 5 weeks (IQR: 4.6–5.3). The median dose per fraction was 2.0 Gy (IQR: 1.8–2.0). Median time from radiotherapy completion to surgery was 6.6 weeks (IQR: 5.9–7.3). Chemotherapy was concurrently associated with radiotherapy in most of patients (*n* = 245, 95.3%). Oral or intravenous 5-FU was mainly used (*n* = 214, 83.3%). The median follow-up was 3.8 years (IQR: 1.9–5.4). Patient, tumor and treatment characteristics are reported in Table 1.

**Table 1:** Patient, tumor and treatment characteristics

Variables	Whole set of patients (*n* = 257, 100%)	Patients with pre-radiation NTL ≤2.8 (*n* = 169, 65.6%)	Patients with pre-radiation NTL >2.8 (*n* = 71, 27.6%)	*p* value
**Clinical data**				
**Median age, years (IQR)**	66.0 (56.4–73.0)	66.8 (56.9–72.7)	63.1 (53.6–75.6)	0.39^†^
<70 years, *n* (%)	173 (67.3)	114 (67.5)	50 (70.4)	0.77^γ^
≥70 years, *n* (%)	84 (32.7)	55 (32.5)	21 (29.6)	
**Gender, *n* (%)**				0.28^γ^
Male	170 (66.1)	110 (65.1)	52 (73.2)	
Female	87 (33.9)	59 (34.9)	19 (26.8)	
**WHO Performance status, *n* (%)**				1^‡^
0–1	251 (97.7)	167 (98.8)	70 (98.6)	
2–3	6 (2.3)	2 (1.2)	1 (1.4)	
**Body mass index, *n* (%)**				0.06^‡^
<18.5	6 (2.3)	2 (1.18)	4 (5.6)	
≥18.5	219 (85.2)	147 (86.98)	57 (80.3)	
Missing data	32	20 (11.8)	10 (14.1)	
**Median follow-up, months (IQR)**	46.1 (23.3–64.7)	52.4 (28.2–66.4)	35.9 (17.9–60.6)	**0.02**^†^
**Biological data (G/L)**				
**Pre-radiation**				
Median neutrophil count	4.05 (3.17–5.35)	3.60 (2.93,4.50)	5.48 (4.59–6.71)	**<0.001^†^**
Median lymphocyte count	1.81 (1.45–2.21)	2.03 (1.69–2.35)	1.48 (1.27–1.7)	**<0.001^†^**
**4 weeks after radiation initiation**				
Median neutrophil count	3.00 (2.31–3.99)	2.71 (2.18–3.45)	3.70 (3.00–4.90)	**<0.001^†^**
Median lymphocyte count	0.70 (0.55–0.90)	0.76 (0.6–0.97)	0.60 (0.47–0.77)	**<0.001^†^**
**Tumor data, *n* (%)**				
**Location**				0.81^γ^
Lower rectum	114 (44.4)	74 (43.8)	32 (45.0)	
Middle rectum	117 (45.5)	76 (45.0)	33 (46.5)	
Upper rectum	26 (10.1)	19 (11.2)	6 (8.5)	
**Tumor Histology**				
Adenocarcinoma	257 (100)	169	71	
**Tumor differenciation**				**0.001^‡^**
Poor	9 (3.5)	4 (2.4)	5 (7.0)	
Moderate	126 (49.0)	76 (45.0)	43 (60.6)	
Well	108 (42.0)	82 (48.5)	17 (23.9)	
Missing data	14 (5.5)	7 (4.1)	6 (8.5)	
**Tumor staging**				0.65^‡^
Stage I	12 (4.7)	7 (4.1)	4 (5.6)	
Stage II	79 (30.7)	56 (33.1)	20 (28.2)	
Stage III	160 (62.3)	102 (60.4)	46 (64.8)	
Unknown (Tx Nx M0)	6 (2.3)	4 (2.4)	1 (1.4)	
**Vascular invasion**				0.42^γ^
Yes	56 (21.8)	33 (19.5)	17 (24.0)	
No	181 (70.4)	125 (74.0)	46 (64.8)	
Missing data	20 (7.8)	11 (6.5)	8 (11.2)	
**Radiation data**				
**Median rectal EQD2, Gy (IQR)**	49.2 (43.9–50.0)	49.2 (43.9–50.0)	49.2 (47.7–50.0)	0.26^†^
**Median dose per fraction, Gy (IQR)**	2.0 (1.8–2.0)	2.0 (1.80–2.0)	2.0 (1.8–2.0)	0.72^†^
**Median duration, days (IQR)**	35 (32–36)	35 (32–36)	35 (32–37)	0.35^†^
**Radiation technique, *n* (%)**				0.83^γ^
3D-CRT	243 (94.5)	160 (94.7)	66 (93.0)	
IMRT	14 (5.5)	9 (5.3)	5 (7.0)	
**Normofractionated regimen, *n* (%)**	237 (92.2)	159 (94.1)	65 (91.5)	0.66^γ^
**Hypofractionated regimen, *n* (%)**	20 (7.8)	10 (5.9)	6 (8.5)	
**Rectal dose reduction, *n* (%)**				0.33^γ^
Yes	127 (49.4)	82 (48.5)	40 (56.4)	
No	130 (50.6)	87 (51.5)	31 (43.6)	
**Time from radiation completion to surgery, days**	46 (41–51)	46 (41–50)	47 (43–54)	0.15^†^
**Chemotherapy data, *n* (%)**				
**Concomitant chemotherapy**	245 (95.4)	164 (97)	67 (94.4)	1^‡^
5-FU	214 (83.3)	143 (84.6)	59 (83.1)	
FOLFOX	19 (7.4)	13 (7.7)	5 (7.1)	
Other	12 (4.7)	8 (4.7)	3 (4.2)	
**No chemotherapy**	12 (4.6)	5 (3.0)	4 (5.6)	

Percentages were calculated based on the population of each column.

Abbreviations: IQR: interquartile range, n: number of patients, 3D-CRT: 3D conformational radiotherapy; IMRT: Intensity-modulated radiotherapy; ^†^Kruskal–Wallis test, ^‡^Fischer test; ^γ^Khi-square test.

### Adjuvant treatments

An adjuvant chemotherapy was performed in 100 patients (38.9%), depending on pre-operative imaging and on pathological findings. Thus, 40 patients (15.5%) with post-operative node involvement on pathological findings underwent adjuvant chemotherapy. Furthermore, 60 patients (23.3%) with node involvement on pre-operative imaging (MRI, TDM, US) but without post-operative node involvement (i.e. complete pathological response on lymph nodes) underwent adjuvant chemotherapy. A total of 28 patients (10.9%) with a theoretical indication of chemotherapy (node involvement) on pathological findings were recused, either because of age, immediate distant progression (metastases) or because of post-operative complications (fistulae).

Oxaliplatin plus infusional 5-fluorouracil (5-FU) and folinic acid (FOLFOX, *n* = 64, 24.9%) or exclusive 5-FU (*n* = 33, 12.8%) were mainly prescribed. Interestingly, only 33 FOLFOX patients (53.1%) and 23 exclusive 5-FU patients (69.7%) received the full dose adjuvant chemotherapy.

### Outcomes

#### Complete resection

Clear surgical margins (*i.e.* complete resection) were achieved in 234 patients (91.1%). A logistic regression was performed in order to assess factors associated with complete resection. The univariate analysis provided potential (*p* < 0.20) predictive factors, with WHO performance status 0–1 (HR = 10.45 CI 95% (1.35–60.06)) and concomitant chemotherapy (HR = 4.54 CI 95% (0.64–20.51), *p* = 0.07). Potential (*p* < 0.20) risk factors of incomplete resection were age ≥70 (HR = 0.39 CI 95% (0.12–1.21), *p* = 0.09), vascular invasion (HR = 0.14 CI 95% (0.03–0.45), *p* = 0.001), and time interval between radiation completion and surgery ≥56 days (HR = 0.34 CI 95% (0.10–1.32), *p* = 0.09). In multivariate analysis, only the vascular invasion was an independent predictive factor of incomplete resection (HR = 0.15 CI 95% (0.04–0.52), *p* = 0.003). Uni- and multi-variate analysis is reported in Table 2.

**Table 2:** Prognostic factors for complete resection (clear surgical margins) based on univariate and multivariate analysis (*n* = 277 patients with 234 complete resections, 13 involved surgical margins, 10 NA)

Variable		Univariate analysis		Multivariate analysis	
	Tested *vs.* Adverse criteria	Logistic regression coefficient (95% CI)	*p*-value	Logistic regression coefficient (95% CI)	*p*-value
Age	≥70 *vs.* <70	0.39 (0.12–1.21)	**0.09**	0.39 (0.11–1.39)	0.15
Gender	Female *vs.* Male	0.56 (0.18–1.80)	0.31		
WHO performance status	0–1 *vs.* 2–3	10.45 (1.35–60.06)	**0.01**	6.31 (0.69–45)	0.07
Body Mass Index	≥18.5 *vs.* <18.5	N.A.	0.99		
Tumor stage	Stage III *vs.* I–II	0.85 (0.22–2.77)	0.79		
Rectal tumor location	Upper *vs.* Middle	0.90 (0.13–17.97)	0.92		
	Lower *vs.* Middle	0.48 (0.13–1.58)	0.24		
Tumor differentiation	Poor *vs.* Well	N.A.	0.99		
	Moderate *vs.* Well	0.82 (0.23–56.11)	0.73		
ypCR	Yes *vs.* No	N.A.	0.99		
Vascular invasion	Yes *vs.* No	0.14 (0.03–0.45)	**0.001**	0.15 (0.04–0.52)	0.003
Time interval between radiation completion and tumor resection	≥56 days vs. <56 days	0.34 (0.10–1.32)	**0.09**		
Pre-Radiation NLR	>2.8 *vs.* ≤2.8	1.27 (0.27–4.59)	0.74		
4WPRN ratio	>1.1 *vs.* ≤1.1	1.04 (0.17–19.82)	0.97		
4WPRL ratio	>0.35 *vs.* ≤0.35	1.14 (0.23–4.81)	0.86		
4WPL ratio	>2.5 *vs.* ≤2.5	2.09 (0.44–7.69)	0.30		
Radiotherapy characteristics	Rectal EQD2 ≥45 Gy *vs.* <45 Gy	1.16 (0.30–3.68)	0.82		
	Pelvis EQD2 ≥40 Gy *vs.* <40 Gy	2.35 (0.12–14.4)	0.44		
	Hypofractionated *vs.* Normofractionated	0.46 (0.11–3.10)	0.34		
Concomitant chemotherapy	Yes *vs.* No	4.54 (0.64–20.51)	**0.07**		

95% CI: 95% confidence interval; ypCR: Pathological complete response (Mandard TRG1); NLR: Neutrophil to Lymphocyte ratio; 4WPRN ratio: “4 weeks” to “pre-radiation” neutrophil ratio; 4WPRL ratio: “4 weeks” to “pre-radiation” lymphocyte ratio; 4WPL ratio: “4 weeks” neutrophil to “4 weeks” lymphocyte ratio. N.A.: Not assessable (no event), typically giving infinity values. EQD2: Equivalent Dose in 2 Grays per fraction; Normofractionated: <2.5 Gy per fraction; Hypofractionated: ≥2.5 Gy per fraction.

All *p*-values ≤ 0.2 in univariate analysis have been tested in multivariate analysis, except correlated variables (correlation with *p* < 0.001). Finally, only bold typed values were tested in multivariate analysis.

#### Pathological response

A complete (Mandard TRG1: *n* = 35, 13.6%) or almost complete pathological response (Mandard TRG2: *n* = 56, 21.8%) was achieved in 91 patients (35.4%). A logistic regression was performed in order to assess the associated factors with good pathological response (Mandard TRG1-2). The univariate analysis provided potential (*p* < 0.20) predictive factors of good response, with WHO performance status 0–1 (HR = 4.63 CI 95% (1.06–20.35), *p* = 0.04) concurrent chemotherapy (HR = 2.22 CI 95% (1.34–3.61), *p* = 0.001), rectal EQD2 ≥45 Gy (HR = 1.41 CI 95% (0.9–2.22), *p* = 0.13), pelvic EQD2 ≥40 Gy (HR = 2.37 CI 95% (1.05–5.27), *p* = 0.02), and 4WPRN ratio > 1.1 (HR = 2.03 CI 95% (0.87–4.74), *p* = 0.10). Potential (*p* < 0.20) predictive factors of bad response were age ≥70 (HR = 0.49 CI 95% (0.33–0.74), *p* < 0.001), stage III tumor (HR = 0.61 CI 95% (0.41–0.92), *p* = 0.02), hypofractionation (HR = 0.51 CI 95% (0.24–1.1), *p* = 0.07) and pre-radiation NLR >2.8 (HR = 0.54 CI 95% (0.29–1.01), *p* = 0.05). In multivariate analysis, age ≥70 years (HR = 0.42 CI 95% (0.22–0.79), *p* = 0.08) and tumor stage III (HR = 0.44 (0.25–0.80), *p* = 0.006) were identified as independent risk factor of poor pathological response. Pre-radiation NTL ratio >2.8 was associated with a close-to-significant higher risk of poor pathological response (HR = 0.53 CI 95% (0.27–1.02), *p* = 0.06). Uni- and multi-variate analysis is reported in Table 3.

**Table 3:** Prognostic factors for good pathological response (Mandard TRG1-2) based on univariate and multivariate analysis (*n* = 257 patients with 35 Mandard TRG1 and 56 Mandard TRG2)

Variable		Univariate analysis		Multivariate analysis	
	Tested *vs.* Adverse criteria	Logistic regression coefficient (95% CI)	*p*-value	Logistic regression coefficient (95% CI)	*p*-value
Age	≥70 *vs.* <70	0.49 (0.33–0.74)	**<0.001**	0.42 (0.22–0.79)	0.008
Gender	Female *vs.* Male	0.92 (0.62–1.39)	0.7		
WHO performance status	0–1 *vs.* 2–3	4.63 (1.05–20.35)	**0.04**		
Body mass index	≥18.5 *vs.* <18.5	2.25 (0.62–8.12)	**0.19**		
Tumor stage	Stage III *vs.* I–II	0.61 (0.41–0.92)	**0.02**	0.44 (0.25–0.80)	0.006
Rectal tumor location	Upper *vs.* Middle	0.86 (0.49–1.87)	0.90		
	Lower *vs.* Middle	0.93 (0.62–1.42)	0.75		
Tumor differentiation	Poor *vs.* Well	0.68 (0.25–1.83)	0.45		
	Moderate *vs.* Well	0.85 (0.55–1.30)	0.44		
Time interval between radiation completion and tumor resection	≥56 days *vs.* <56 days	0.91 (0.55–1.52)	0.73		
Pre-Radiation NLR	>2.8 *vs.* ≤2.8	0.54 (0.29–1.01)	**0.05**	0.53 (0.27–1.02)	0.06
4WPRN ratio	>1.1 *vs.* ≤1.1	2.03 (0.87–4.74)	**0.10**		
4WPRL ratio	>0.35 *vs.* ≤0.35	0.68 (0.37–1.24)	**0.21**		
4WPL ratio	>2.5 *vs.* ≤2.5	1.51 (0.67–3.38)	**0.32**		
Radiotherapy characteristics	Rectal EQD2 ≥45 Gy *vs.* <45 Gy	1.41 (0.9–2.22)	**0.13**	1.62 (0.83–3.16)	0.16
	Pelvis EQD2 ≥40 Gy *vs.* <40 Gy	2.37 (1.05–5.27)	**0.02**		
	Hypofractionated *vs.* Normofractionated	0.51 (0.24–1.1)	**0.07**		
Concomitant chemotherapy	Yes *vs.* No	2.22 (1.34–3.61)	**0.001**		

95% CI: 95% confidence interval; ypCR: Pathological complete response (Mandard TRG1); NLR: Neutrophil to Lymphocyte ratio; 4WPRN ratio: “4 weeks” to “pre-radiation” neutrophil ratio; 4WPRL ratio: “4 weeks” to “pre-radiation” lymphocyte ratio; 4WPL ratio: “4 weeks” neutrophil to “4 weeks” lymphocyte ratio. N.A.: Not assessable (no event), typically giving infinity values. EQD2: Equivalent Dose in 2 Grays per fraction; Normofractionated: <2.5 Gy per fraction; Hypofractionated: ≥2.5 Gy per fraction.

All *p*-values ≤ 0.2 in univariate analysis have been tested in multivariate analysis, except correlated variables (correlation with *p* < 0.001). Finally, only bold typed values were tested in multivariate analysis.

#### Local recurrence

At the end of follow-up, 8 patients (3.1%) experienced local relapse after radiotherapy in the whole set of patients. A logistic regression was performed in order to assess the associated factors with local recurrence. The univariate analysis provided potential (*p* < 0.20) predictive protective factors, with complete tumor resection (HR = 0.05 CI 95% (0.01–0.29), *p* < 0.001) and tumor moderate differentiation (HR = 0.04 CI 95% (0.05–1.57), *p* = 0.19). Potential (*p* < 0.20) predictive risk factors were also identified with pre-radiation NLR >2.8 (HR = 4.98 CI 95% (0.95–36.5), *p* = 0.07). In multivariate analysis, complete tumor resection (HR = 0.05 CI 95% (0.01–0.29), *p* < 0.001) and well differentiated tumors (HR = 0.04 (0.01–0.5), *p* = 0.04) were identified as independent protective factors of local recurrence. Pre-radiation NLR >2.8 was identified as an independent risk factor of local recurrence (HR = 14.7 CI 95% (1.53–334.3), *p* = 0.03). Uni- and multi-variate analysis is reported in Table 4.

**Table 4:** Prognostic factors for local tumor recurrence based on univariate and multivariate analysis (*n* = 257 patients with 8 local recurrences)

Variable		Univariate analysis		Multivariate analysis	
	Tested *vs.* Adverse criteria	Logistic regression coefficient (95% CI)	*p*-value	Logistic regression coefficient (95% CI)	*p*-value
Age	≥70 *vs.* <70	2.11 (0.49–9.14)	0.29		
Gender	Female *vs.* Male	2.0 (0.46–8.65)	0.34		
WHO performance status	0–1 *vs.* 2–3	N.A.	N.A.		
Body mass index	≥18.5 *vs.* <18.5	N.A.	0.99		
Tumor stage	Stage III *vs.* I–II	0.95 (0.23–4.70)	0.94		
Rectal tumor location	Upper *vs.* Middle	N.A.	0.99		
	Lower *vs.* Middle	1.74 (0.42–8.66)	0.45		
Tumor differentiation	Well *vs.* Poor	N.A.	0.99	N.A.	0.99
	Well *vs.* Moderate	0.33 (0.05–1.57)	**0.19**	0.04 (0.001–0.50)	0.04
Complete tumor resection	Yes *vs.* No	0.07 (0.02–0.39)	**0.001**	0.02 (0.001–0.27)	0.004
ypCR	Yes *vs.* No	0.98 (0.05–6.0)	0.97		
Vascular invasion	Yes *vs.* No	1.08 (0.16–4.85)	0.93		
Time interval between radiation completion and tumor resection	≥56 days *vs*. <56 days	N.A.	0.93		
Pre-Radiation NLR	>2.8 *vs.* ≤2.8	4.98 (0.95–36.5)	**0.07**	14.7 (1.53–334.30)	0.03
4WPRN ratio	>1.1 *vs.* ≤1.1	N.A.	0.99		
4WPRL ratio	>0.35 *vs.* ≤0.35	0.539 (0.06–4.57)	0.54		
4WPL ratio	>2.5 *vs.* ≤2.5	N.A.	0.99		
Radiotherapy characteristics	Rectal EQD2 ≥45 Gy *vs.* <45 Gy	0.61 (0.15–3.05)	0.51		
	Pelvis EQD2 ≥40 Gy *vs.* <40 Gy	N.A.	0.99		
	Hypofractionated *vs.* Normofractionated	N.A.	0.99		
Concomitant chemotherapy	Yes *vs.* No	0.32 (0.05–6.32)	0.31		

95% CI: 95% confidence interval; ypCR: Pathological complete response (Mandard TRG1); NLR: Neutrophil to Lymphocyte ratio; 4WPRN ratio: “4 weeks” to “pre-radiation” neutrophil ratio; 4WPRL ratio: “4 weeks” to “pre-radiation” lymphocyte ratio; 4WPL ratio: “4 weeks” neutrophil to “4 weeks” lymphocyte ratio. N.A.: Not assessable (no event), typically giving infinity values. EQD2: Equivalent Dose in 2 Grays per fraction; Normofractionated: <2.5 Gy per fraction; Hypofractionated: ≥2.5 Gy per fraction.

All *p*-values ≤ 0.2 in univariate analysis have been tested in multivariate analysis, except correlated variables (correlation with *p* < 0.001). Finally, only bold typed values were tested in multivariate analysis.

#### Progression-free survival (PFS)

At last follow-up, 8 patients (3.1%) experienced local relapse, 38 (14.8%) experienced metastases and 45 (17.5%) had died. Median PFS was therefore not reached. Two-year and 5-year PFS were 79.4% (CI95%: 74.3–84.9) and 67.7% (CI 95%: 61.3–74.3), respectively. A logistic regression (univariate and multivariate analysis) was performed in order to assess the associated factors with PFS. The univariate analysis provided potential (*p* < 0.20) predictive factors of PFS, with female gender (HR = 0.65 CI 95% (0.38–1.12), *p* = 0.12), complete tumor resection (HR = 0.19 CI 95% (0.09–0.37), *p* < 0.001), complete (*i.e.* Mandard TRG1) pathological response (HR = 0.53 CI 95% (0.21–1.21), *p* = 0.18), rectal EQD2 ≥ 45Gy (HR = 0.71 CI 95% (0.43–1.18), *p* = 0.19), and concomitant chemotherapy (HR = 0.45 CI 95% (0.18–1.13), *p* = 0.08). Potential (*p* < 0.20) predictive factors of tumor progression were age ≥70 years (HR = 2.73 CI 95% (1.68–4.43), *p* < 0.001), low tumor location (HR = 1.71 CI 95% (1.02–2.88), *p* = 0.04), time interval between radiation completion and surgery ≥56 days (HR = 1.59 CI 95% (0.53–7.0), *p* = 0.13), and pre-radiation NLR > 2.8 (HR = 2.29 CI 95% (1.35–3.90), *p* = 0.002). In multivariate analysis, age ≥70 (HR = 2.05 CI 95% (1.18–3.54), *p* = 0.01), and pre-radiation NLR >2.8 (HR = 2.21 CI 95% (1.26–3.86), *p* = 0.006) were independent predictive factor of progression. Complete tumor resection (HR = 0.29 CI 95% (0.13–0.64), *p* = 0.002) was the only independent protective factor. Uni- and multi-variate analysis is reported in Table 5.

**Table 5:** Prognostic factors for progression-free survival based on univariate and multivariate analysis (*n* = 257 patients: 8 local recurrences, 38 metastatic relapses, 45 deaths)

Variable		Univariate analysis		Multivariate analysis	
	Tested *vs.* Adverse criteria	Hazard ratio (95% CI)	*p*-value (log-rank test)	Hazard ratio (95% CI)	*p*-value (cox model)
Age	≥70 *vs.* <70	2.73 (1.68–4.43)	**<0.001**	2.05 (1.18–3.54)	0.01
Gender	Female *vs.* Male	0.65 (0.38–1.12)	**0.12**		
WHO performance status	0–1 *vs.* 2–3	1.23 (0.30–5.04)	0.77		
Body mass index	≥18.5 *vs.* <18.5	0.60 (0.14–2.47)	0.48		
Tumor stage	Stage III *vs.* I–II	1.34 (0.79–2.26)	0.28		
Rectal tumor location	Upper *vs.* Middle	0.84 (0.32–2.22)	0.73		
	Lower *vs.* Middle	1.71 (1.02–2.88)	0.04		
Tumor differentiation	Poor *vs.* Well	0.74 (0.56–1.57)	0.69		
	Moderate *vs.* Well	0.94 (0.18–3.14)	0.82		
Complete tumor resection	Yes *vs.* No	0.19 (0.09–0.37)	**<0.001**	0.29 (0.13–0.64)	0.002
ypCR	Yes *vs.* No	0.53 (0.21–1.32)	**0.18**		
Vascular invasion	Yes *vs.* No	1.41 (0.81–2.44)	0.22		
Time interval between radiation completion and tumor resection	≥56 days vs. <56 days	1.59 (0.86–2.91)	0.13		
Pre-Radiation NLR	>2.8 *vs.* ≤2.8	2.29 (1.35–3.90)	**0.002**	2.21 (1.26–3.86)	0.006
4WPRN ratio	>1.1 *vs.* ≤1.1	0.71 (0.28–1.79)	0.47		
4WPRL ratio	>0.35 *vs.* ≤0.35	1.42 (0.75–2.68)	0.27		
4WPL ratio	>2.5 *vs.* ≤2.5	1.20 (0.59–2.45)	0.61		
Radiotherapy characteristics	Rectal EQD2 ≥45 Gy *vs.* <45 Gy	0.71 (0.43–1.18)	**0.19**	0.61 (0.35–1.08)	0.09
	Pelvis EQD2 ≥40 Gy *vs.* <40 Gy	1.35 (0.33–5.55)	0.67		
	Hypofractionated *vs.* Normofractionated	1.44 (0.65–3.15)	0.36		
Concomitant chemotherapy	Yes *vs.* No	0.45 (0.18–1.13)	**0.09**		

95% CI: 95% confidence interval; ypCR: Pathological complete response (Mandard TRG1); NLR: Neutrophil to Lymphocyte ratio; 4WPRN ratio: “4 weeks” to “pre-radiation” neutrophil ratio; 4WPRL ratio: “4 weeks” to “pre-radiation” lymphocyte ratio; 4WPL ratio: “4 weeks” neutrophil to “4 weeks” lymphocyte ratio. N.A.: Not assessable (no event), typically giving infinity values. EQD2: Equivalent Dose in 2 Grays per fraction; Normofractionated: <2.5 Gy per fraction; Hypofractionated: ≥2.5 Gy per fraction.

All *p*-values ≤ 0.2 in univariate analysis have been tested in multivariate analysis, except correlated variables (correlation with *p* < 0.001). Finally, only bold typed values were tested in multivariate analysis.

#### Overall Survival (OS)

**Figure 1:**
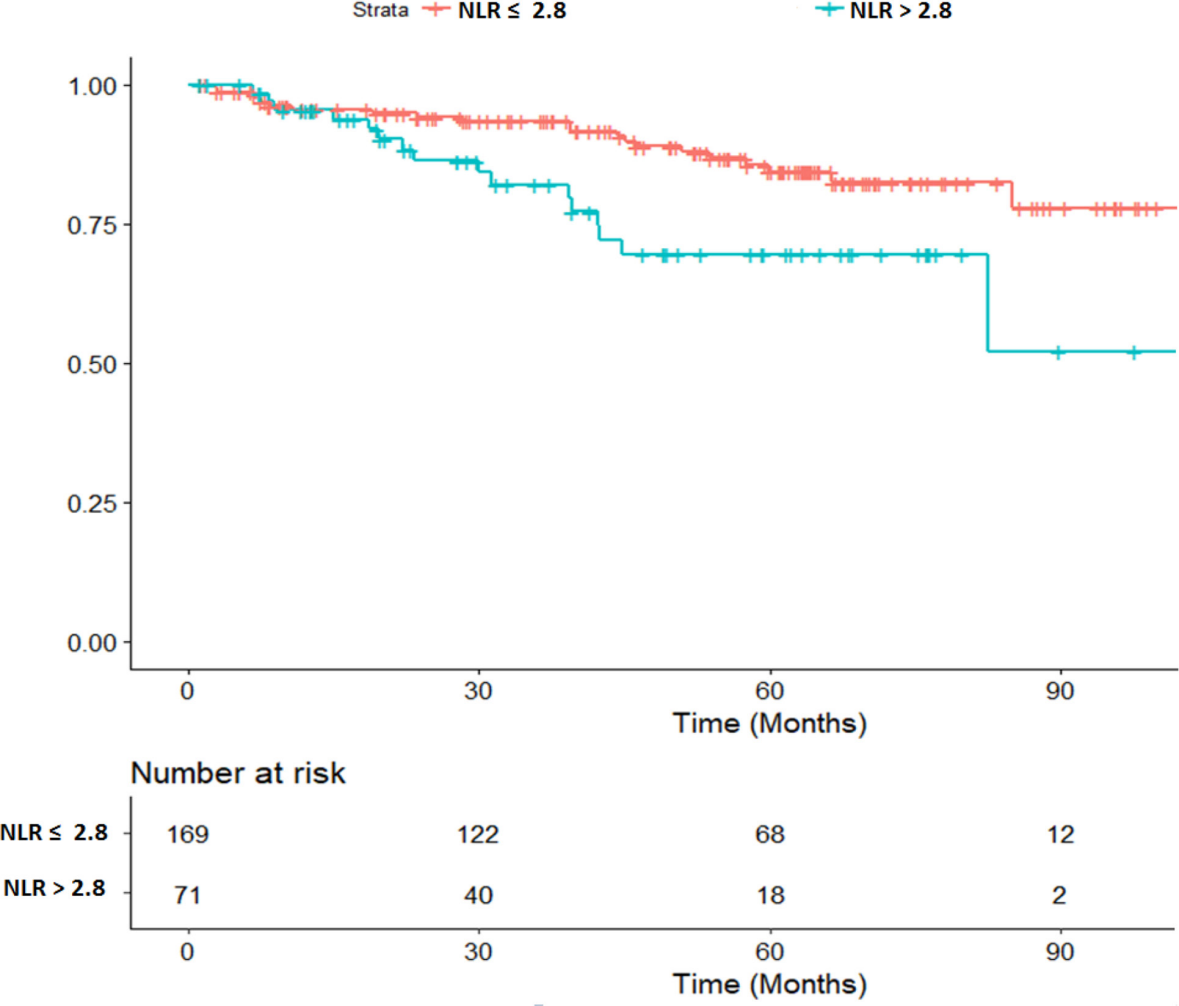
Overall survival depending on the pre-radiation Neutrophil to Lymphocyte Ratio (NLR). (HR 2.23 CI 95% (1.14–2.36), *p* = 0.02)

At last follow-up, 45 patients (17.5%) had died. Median OS was therefore not reached. Two-year and 5-year OS were 91.4% (CI 95%: 87.9–95.1) and 77.5% (CI 95%: 71.5–84.1), respectively. A logistic regression (univariate and multivariate analysis) was performed in order to assess the associated factors with OS. The univariate analysis provided potential (*p* < 0.20) predictive factors of OS, with complete pathological response (HR = 0.56 CI 95% (0.28–1.11), *p* = 0.10) and concurrent chemotherapy (HR = 0.48 CI 95% (0.34–0.68), *p* < 0.001). Potential (*p* < 0.20) predictive factors of death were age ≥70 (HR = 3.67 CI 95% (2.52–5.34), *p* < 0.001), WHO performance status 2–3 (HR = 2.02, CI 95% (1.03–3.99), *p* = 0.04), stage III tumors (HR = 1.51 CI 95% (1.04–2.20), *p* = 0.03), poor tumor differentiation (HR = 1.70 CI 95% (0.81–3.60, *p* = 0.16), vascular invasion (HR = 1.84 CI 95% (1.25–2.72), *p* = 0.002), time interval between radiation completion and surgery ≥56 days (HR = 1.78 CI 95% (1.19–2.68), *p* = 0.005) and pre-radiation NLR >2.8 (HR = 2.30 CI 95% (1.20–4.43), *p* = 0.01). In multivariate analysis, age ≥70 (HR = 2.64 CI 95% (1.35–5.17), *p* = 0.004), stage III tumors (HR = 2.48 CI 95% (1.15–5.38), *p* = 0.02) and pre-radiation NLR >2.8 (HR = 2.23 CI 95% (1.14–2.36), *p* = 0.02) were identified as independent predictive factors of death. Uni- and multi-variate analysis is reported in Table 6. Overall survival depending on pre-radiation NLR is depicted in Figure 1.

**Table 6:** Prognostic factors for Overall Survival based on univariate and multivariate analysis (*n* = 257 patients with 45 deaths)

Variable		Univariate analysis		Multivariate analysis	
	Tested *vs.* Adverse criteria	Hazard ratio (95% CI)	*p*-value (log-rank test)	Hazard ratio (95% CI)	*p*-value (cox model)
Age	≥70 *vs.* <70	3.67 (2.52–5.34)	**<0.001**	2.64 (1.35–5.17)	0.004
Gender	Female *vs.* Male	0.81 (0.57–1.17)	0.27		
WHO performance status	2–3 *vs.* 0–1	2.02 (1.03–3.99)	0.04		
Body mass index	≥18.5 *vs.* <18.5	0.74 (0.30–1.83)	0.52		
Tumor stage	Stage III *vs.* I–II	1.51 (1.04–2.20)	**0.03**	2.48 (1.15–5.38)	0.02
Rectal tumor location	Upper *vs.* Middle	1.13 (0.65–1.96)	0.66		
	Lower *vs.* Middle	1.19 (0.82–1.73)	0.35		
Tumor differentiation	Poor *vs.* Well	1.70 (0.81–3.60)	**0.16**		
	Moderate *vs.* Well	1.10 (0.75–1.60)	0.64		
Complete tumor resection	Yes *vs.* No	0.33 (0.21–0.53)	<0.001		
ypCR	Yes *vs.* No	0.56 (0.28–1.11)	**0.10**		
Vascular invasion	Yes *vs.* No	1.84 (1.25–2.72)	0.002		
Time interval between RT completion and tumor resection	≥56 days vs. <56 days	1.78 (1.19–2.68)	**0.005**		
Pre-Radiation NLR	>2.8 *vs.* ≤2.8	2.30 (1.20–4.43)	**0.01**	2.23 (1.14–2.36)	0.02
4WPRN ratio	>1.1 vs ≤1.1	0.78 (0.27–2.21)	0.64		
4WPRL ratio	>0.35 vs ≤0.35	1.09 (0.52–2.29)	0.81		
4WPL ratio	>2.5 vs ≤2.5	1.5 (0.63–3.57)	0.363		
Radiotherapy characteristics	Rectal EQD2 ≥45 Gy *vs.* <45 Gy	0.99 (0.69–1.43)	0.97		
	Pelvis EQD2 ≥40 Gy *vs.* <40 Gy	0.94 (0.56–1.57)	0.81		
	Hypofractionated *vs.* Normaofractionated	1.00 (0.58–1.70)	0.99		
Concomitant chemotherapy	Yes *vs.* No	0.48 (0.34–0.68)	<0.001		

95% CI: 95% confidence interval; ypCR: Pathological complete response (Mandard TRG1); NLR: Neutrophil to Lymphocyte ratio; 4WPRN ratio: “4 weeks” to “pre-radiation” neutrophil ratio; 4WPRL ratio: “4 weeks” to “pre-radiation” lymphocyte ratio; 4WPL ratio: “4 weeks” neutrophil to “4 weeks” lymphocyte ratio. N.A.: Not assessable (no event), typically giving infinity values. EQD2: Equivalent Dose in 2 Grays per fraction; Normofractionated: <2.5 Gy per fraction; Hypofractionated: ≥2.5 Gy per fraction.

All *p*-values ≤0.2 in univariate analysis have been tested in multivariate analysis, except correlated variables (correlation with *p* < 0.001). Finally, only bold typed values were tested in multivariate analysis.

## DISCUSSION

Independent predictive factors of efficacy were retrospectively analyzed in a large cohort of RC patients in the present study, with a special focus on peripheral immune biomarkers. Pre-radiation NLR was an independent predictive factor of local relapse, of PFS, of OS and was marginally associated with complete pathological response, suggesting a value as a possible biomarker of radio-sensitivity. Combined index of peripheral neutrophil and lymphocyte counts (and especially NLR) have been shown to correlate with survival outcomes in numerous malignancies treated with definitive chemoradiation including cervical [16], lung [17] and gastrointestinal cancers [5–7, 9, 13, 14], suggesting that NLR is not specifically related to rectal (or certainly any other type of) cancer. However, the present study is one of the largest series of rectal cancer patients in literature, since numerous previous publications mixed colon and rectal cancer patients.

The NLR is thought to reflect the host’ systemic inflammatory response and immunologic status, through the balance between pro- and anti-tumor activities of immune cells. On the one hand, neutrophils are suggested to induce a pro-tumor effect on the local microenvironment, secreting cytokines recruiting inflammatory cells, favoring angiogenesis [18], invasion [19], tumor growth, and suppressing the adaptive immune response [20] especially through the inhibition of natural killer cells and activated T cells [21]. To our best knowledge, no pre-clinical or clinical study specifically examined the underlying biological mechanisms associated with neutrophils in rectal cancer patients. However, a study was recently conducted in a large series of cervical cancer patients, resulting in appealing theories linking tumor-related neutrophilia and poor outcomes [22]. Neutrophilia (Neutrophil count > 7.5 G/L) at the initial diagnosis was clearly shown to be an independent predictor of compromised survival, indeed. Furthermore, a strong correlation was observed between G-CSF immunoreactivity in patients’ tumors and peripheral neutrophil count, and between G-CSF immunoreactivity and decreased survival, suggesting that the granulopoiesis induced by tumor-derived G-CSF might be the root of tumor-related neutrophilia that finally correlates with poor outcomes. Interestingly, patient outcomes were supported by strong pre-clinical data. In murine models, subjects inoculated with cancer cells expressing tumor-derived GCSF were reported to significantly develop more myeloid-derived suppressor cells (a subpopulation enhancing tumor progression by stimulating tumor angiogenesis, metastasis and immune suppression) in blood, bone marrow and tumor, resulting in more aggressive tumors. Interestingly, cancer cells expressing G-GSF were significantly less sensitive to radiation tha t control cancer cells, through yet unclear processes [22]. On the other hand, lymphocytes are thought to have an anti-tumor effect, since it is now known for a long time that tumor infiltration of CD4+ and CD8+ cells is associated with improved survival in RC patients [23, 24]. Furthermore, lymphocytes are suggested to play a crucial role in cancer immune surveillance and immunoediting [25], probably explaining why patients with high lymphocyte densities achieve long term local and distant control after resection [23]. Elevated NLR could therefore reflect the predominance of neutrophils’ pro-tumor activity and/or the weakness of the lymphocytes’ anti-tumor immune response. The exact biological phenomena underlying the fact that patients with elevated NLR show unfavorable local and overall outcome are still investigated, but taken together, all results seem to indicate that the NLR is a simple and robust biomarker for risk stratification in locally advanced RC patients, with a threshold ranging from 2 to 5 in literature [26, 27]. Although the relation between peripheral immune biomarkers and radiation sensitivity is not yet established, the influence of tumor micro-environment and radiosensitivity has been clearly demonstrated [28–30]. The present findings support the hypothesis that immune cell counts, which presumably reflect host conditions, may critically affect responsiveness to radiation.

These results are of primary interest since conventional prognostic factors currently fail to identify patients with the highest risk of local and metastatic recurrence, with more than 40% of patients experiencing lethal relapse. The present findings could therefore contribute to isolate a subpopulation eligible for treatment intensification (radiation dose escalation and/or chemotherapy optimization) at the outset. The current standard of care for locally advanced RC is a neoadjuvant chemoradiotherapy [31, 32] followed by definitive resection. A post-operative chemotherapy is indicated only when bad prognosis factors are identified after rectal surgery, inducing favorable results on local recurrence and PFS [1] but disappointing results on OS [1, 33–35]. Post-operative chemotherapy failed to improve OS in all randomized trials using pre-operative chemoradiation followed by total mesorectal excision, indeed. However, less than 50% of eligible patients received the full dose and course without interruption or delays owing to postoperative complications, delayed recovery, or interference caused by ostomy closure. For the same reasons, nearly a third of patients with locally advanced RC never started the indicated adjuvant chemotherapy. In the present study, similar orders of magnitude were reported, with more than a patient out of ten not undergoing the indicated adjuvant chemotherapy, and with about on patient out of two who could not receive the full dose. Therefore, moving the setting of chemotherapy from adjuvant to neoadjuvant is carefully considered, since it is expected to bring a better safety (better bone marrow function, no stoma resulting in less treatment delays and dose reduction because of stoma complications, shorter duration of stoma use) and maybe a better efficacy (early treatment of the micrometastatic disease, better local response making the surgery unnecessary in selected patients). Finally, there is growing evidence suggesting that patients with bad prognosis factors could benefit from a neoadjuvant chemotherapy performed before chemoradiation [36, 37]. Currently known factors of high risk of distant relapse (node involvement, mesorectal invasion, positive circumferential resection margin, advanced T stage) were recently hypothesized to possibly indicate a preoperative chemotherapy. Clinical trials are currently conducted to explore the efficacy of this management, through the RAPIDO (NCT01558921), PROSPECT (NCT01515787), NRG-GI002 (NCT02921256), BACCHUS (NCT01650428) and Rectal Cancer Consortium (NCT02008656) clinical trials. However, since current RC prognosis factors are thought to be not discriminant enough, and since many of them are only available after surgery completion or state-of-the-art MRI, new simple pre-treatment biomarkers are awaited in order to better predict which patients will be non-responders to chemoradiation, and should therefore experience neo-adjuvant chemotherapy. In this context, the pre-treatment NLR seem to be able to bring crucial information on prognostication before treatment initiation, being an independent predictive factor of local recurrence, PFS and OS. Although the retrospective nature of the present study is a limitation, these results should encourage the prospective exploration of peripheral immune biomarkers in RC patients, in order to validate its metrological characteristics.

## MATERIALS AND METHODS

A retrospective study was conducted at the Lucien Neuwirth comprehensive cancer care center (Saint Priest en Jarez, France). The institutional review board approved the study, which was conducted in compliance with the Helsinki Declaration.

### Patient population and biology

Medical records of all consecutive patients undergoing a pre-operative radiotherapy for a non-metastatic RC between 2004 and 2015 were retrospectively reviewed. Only patients with available biological data (*i.e.* neutrophil and/or lymphocyte counts) were selected. Patient characteristics (age, sex, WHO performance status, body mass index (BMI)), tumor histology and staging, radiotherapy characteristics, administered chemotherapy, complete sterilization of the operative specimen (ypCR), complete tumor resection (R0), local recurrence, progression-free survival (PFS) and overall survival (OS) were also studied. Regarding pathological response, the Mandard tumor regression grade (TRG) was also used [38], classifying patients into “good responders” (complete pathological response with no viable cancer cell (TRG1, *i.e.* ypCR) or almost complete pathological response with rare cancer cells that might be still viable (TRG2)) and into “poor responders” (Mandard TGR3-5).

The absolute neutrophil and lymphocyte counts were obtained from samples collected within 30 days prior to radiotherapy initiation and during the fourth week after radiation initiation. Neutrophil to Lymphocyte Ratio (NLR) was obtained by dividing absolute pre-radiation neutrophil to lymphocyte count. “Four-weeks” to “pre-radiation” neutrophil (4WPRN) ratio was obtained by dividing absolute 4-weeks neutrophil count to pre-radiation neutrophil count. “Four-weeks” to “pre-radiation” lymphocyte (4WPRL) ratio was obtained by dividing absolute 4-weeks lymphocyte count to pre-radiation lymphocyte count. “Four weeks” neutrophil to lymphocyte ratio (4WPL) was obtained by dividing absolute 4-week neutrophil count to 4-weeks lymphocyte count. The Contal and Q’Quigley method was used to find optimal threshold values for NLR, 4WPRN, 4WPRL and 4WPL. In the whole set of patient, a NLR cut-off of 2.8 was found to have the highest log-rank statistic (regarding OS). For 4WPRN, 4WPRL and 4WPL ratios, cut-offs points of 1.1, 0.35 and 2.5 were identified, respectively.

### Treatment definition

#### Radiation therapy

Patients were treated in supine position, and immobilized using leg-positioning foamed wedges. CT-scan images were acquired without contrast agent infusion with a slice thickness of 2.5 mm. Plans were contoured and calculated using the Eclipse treatment planning system (Varian Medical Systems, Palto Alto, CA, USA). Gross tumor volume (GTV), clinical tumor volume (CTV), planning tumor volume (PTV) and organs at risk (OAR) were delineated based on planning-CT. Their definition evolved with the availability and development of CT-scan and MRI, and with the delineation guidelines’ editions. In each case, treatment plans were optimized according to dose limits for OAR and constraints for volume coverage i.e. PTV should receive 95% to 107% of the prescribed dose. Rectal equivalent 2 Gy (EQD2) dose was calculated using the EQD2 formula provided by Fowler [39] and α/β = 6.2 [40].

#### Surgery

A curative rectal resection (Total Mesorectal Excision) was systematically performed after radiotherapy completion.

### Evaluation of efficacy

Follow-up was calculated from the initiation of radiotherapy. After radiotherapy completion, patients were assessed for efficacy every 3 months by surgeons and oncologists during the first two years and every 6 months later, with clinical examination and alternation of chest/abdomen/pelvis- CT-scan and chest radiography and abdominal ultrasound.

### Statistical analysis

Median values were given with the interquartile range (IQR: 25%–75%) or with the range (Min-Max). Chi-2 test, Kruskal–Wallis test or Fisher test were performed to compare patient characteristics distribution. PFS was defined as the time from the date of radiotherapy initiation to the date of clinical and/or radiological RC progression. OS was defined as the time from the date of radiotherapy initiation to the date of death or the last follow-up. PFS and OS were estimated with the Kaplan–Meier method. Median survivals were compared using log-rank test. A Cox proportional hazards model was used to test the interaction between data on survival or on local control and treatment or patient characteristics. The multivariate analysis was performed using a Cox multivariate analysis based on the significant -or close-to-significance (*p* < 0.2)- factors. The multivariate model was refined using the AIC criteria. All *p* values were nominal without adjustment for multiple testing. Significance was defined by *p* < 0.05. Statistical analyses were processed with R-3·4·0 (R Core Team. R Foundation for Statistical Computing, Vienna, Austria).

## CONCLUSIONS

Pre-radiation NLR is a simple and robust biomarker for risk stratification in locally advanced RC patients undergoing pre-operative radiotherapy. Indeed, it was correlated with local recurrence, PFS and OS in multivariate analysis. NLR was marginally associated with complete pathological response, suggesting a value as a possible biomarker of radio-sensitivity. These results could lead the way to the accurate selection of the subpopulation of RC patients eligible to neoadjuvant chemotherapy. Although NLR is not a quite new marker, with more than 100 studies on colorectal adenocarcinoma available in MEDLINE, the present data corroborates previous results, in one of the largest cohort of RC Caucasian patients treated with pre-TME radiation. A study examining the underlying biological mechanisms connecting LNR with poor prognosis and local radioresistance in RC patients is still to be carried out. Finally, the retrospective nature of the study induced unavoidable statistical biases that could only be controlled by prospective explorations. Therefore, statistical correlations that were presently suggested should be corroborated by future prospective studies.

## Abbreviations

AIC: Akaike Information Criteria
BMI: Body Mass Index
HR: Hazard Ratio
IQR: Interquartile Range
NLR: Neutrophil to Lymphocyte Ratio
MRI: Magnetic Resonance Imaging
OS: Overall Survival
PFS: Progression-Free Survival
RC: Rectal Cancer
R0: complete tumor regression
TRG: Tumor Regression Grade
ypCR: complete sterilization of the operative specimen
WHO: World Health Organization
4WPRN ratio: “Four-weeks” to “pre-radiation” neutrophil ratio
4WPRL ratio: “Four-weeks” to “pre-radiation” lymphocyte ratio
4WPL ratio: “Four weeks” neutrophil to lymphocyte ratio.
